# Supplementary material for: Investigating microbial population structure and function in the chicken caeca and large intestine over time using metagenomics
Source: BMC Res Notes. 2025 Aug 15;18:355. doi: 10.1186/s13104-025-07441-7 (PMC12357378; doi:10.1186/s13104-025-07441-7)
Supplement: Supplementary file 2 — Recovered nutrient cycles including Carbon, Nitrogen, and Sulphur cycles for all the metagenomic assembled genomes given as PDF images, and identifiable through bin numbers. [file 13104_2025_7441_MOESM2_ESM.zip › Supplementary Data2/bin.105.draw_nitrogen_cycle_single.pdf]

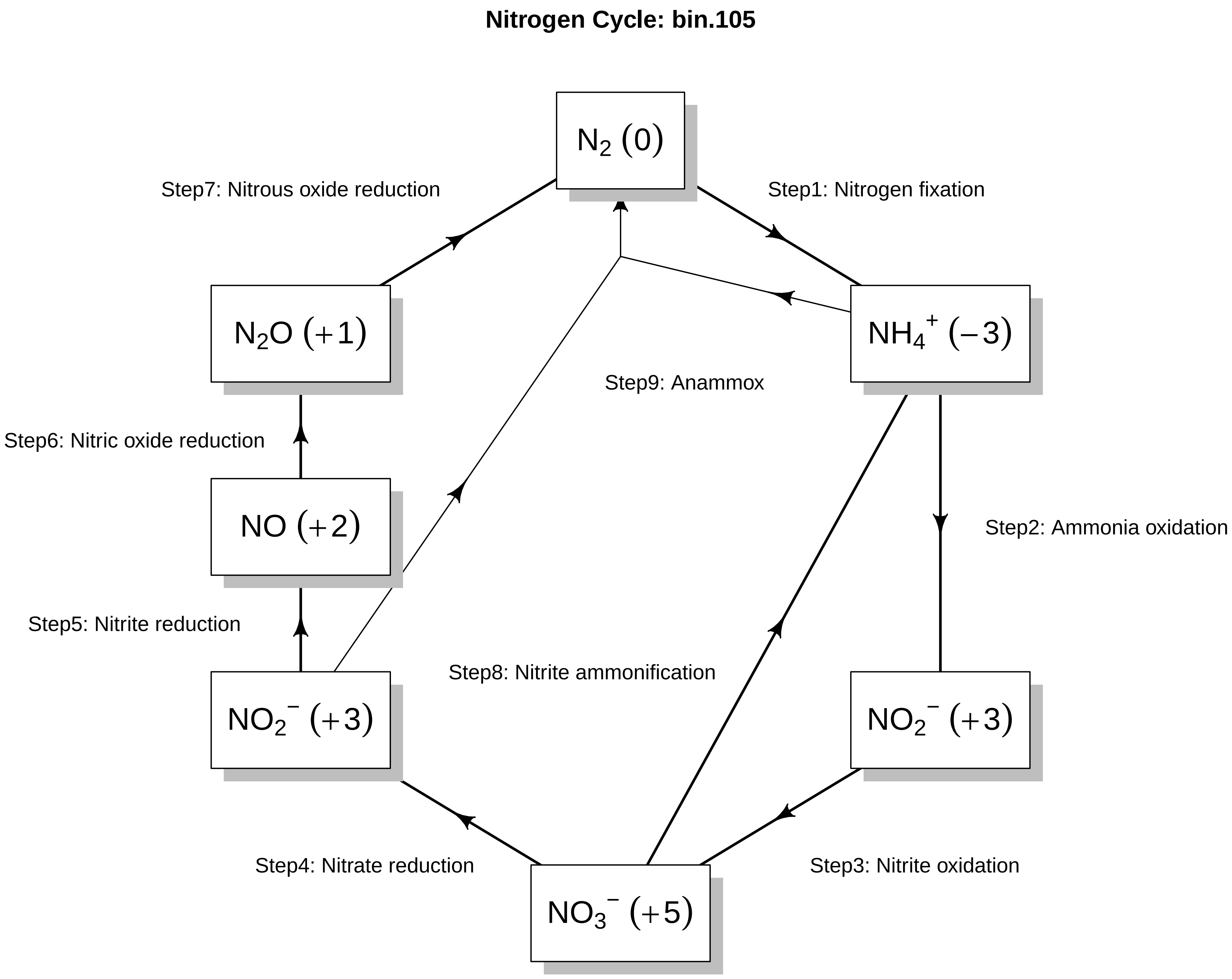
Nitrogen Cycle: bin.105
N_2 (0)
Step7: Nitrous oxide reduction
Step1: Nitrogen fixation
N_2O (+1)
NH_4^+ (−3)
Step9: Anammox
Step6: Nitric oxide reduction
NO (+2)
Step2: Ammonia oxidation
Step5: Nitrite reduction
Step8: Nitrite ammonification
NO_2^- (+3)
NO_2^- (+3)
Step4: Nitrate reduction
Step3: Nitrite oxidation
NO_3^- (+5)
